# Identification of Genetic Variation between Obligate Plant Pathogens *Pseudoperonospora cubensis* and *P*. *humuli* Using RNA Sequencing and Genotyping-By-Sequencing

**DOI:** 10.1371/journal.pone.0143665

**Published:** 2015-11-23

**Authors:** Carly F. Summers, Colwyn M. Gulliford, Craig H. Carlson, Jacquelyn A. Lillis, Maryn O. Carlson, Lance Cadle-Davidson, David H. Gent, Christine D. Smart

**Affiliations:** 1 Plant Pathology and Plant-Microbe Biology Section, School of Integrative Plant Science, Cornell University, Geneva, New York, United States of America; 2 Cornell Laboratory for Accelerator-based Sciences and Education, Cornell University, Ithaca, New York, United States of America; 3 Horticulture Section, School of Integrative Plant Science, Cornell University, Geneva, New York, United States of America; 4 United States Department of Agriculture Agricultural Research Service, Grape Genetics Research Unit, Geneva, New York, United States of America; 5 United States Department of Agriculture Agricultural Research Service, Forage Seed and Cereal Research Unit and Department of Botany and Plant Pathology, Oregon State University, Corvallis, Oregon, United States of America; The University of Hong Kong, HONG KONG

## Abstract

RNA sequencing (RNA-seq) and genotyping-by-sequencing (GBS) were used for single nucleotide polymorphism (SNP) identification from two economically important obligate plant pathogens, *Pseudoperonospora cubensis* and *P*. *humuli*. Twenty isolates of *P*. *cubensis* and 19 isolates of *P*. *humuli* were genotyped using RNA-seq and GBS. Principle components analysis (PCA) of each data set showed genetic separation between the two species. Additionally, results supported previous findings that *P*. *cubensis* isolates from squash are genetically distinct from cucumber and cantaloupe isolates. A PCA-based procedure was used to identify SNPs correlated with the separation of the two species, with 994 and 4,231 PCA-correlated SNPs found within the RNA-seq and GBS data, respectively. The corresponding unigenes (n = 800) containing these potential species-specific SNPs were then annotated and 135 putative pathogenicity genes, including 3 effectors, were identified. The characterization of genes containing SNPs differentiating these two closely related downy mildew species may contribute to the development of improved detection and diagnosis strategies and improve our understanding of host specificity pathways.

## Introduction

The downy mildews are obligate biotrophic pathogens of flowering plants [1]. Elucidating the taxonomy among downy mildew species is especially challenging due to the obligate nature of these pathogens. Where biological separation of species has traditionally relied upon observations of morphology, downy mildew pathogens grow within host tissue, leaving only reproductive structures for observation [2]. Because the appearance of these structures, as well as host symptoms, may vary widely depending on host substrate and environment, morphological characters are not always useful for differentiating species of downy mildews [2]. Host specificity studies have also been used in the past to differentiate species of downy mildews [2,3] but suffer from limitations because of the overlapping host range of certain organisms. Today, both morphological and host range studies have been replaced by phylogenetic analyses for species designation [4]. However, the downy mildews, and oomycetes in general, are often not easily differentiated with highly conserved DNA sequences such as ribosomal RNA genes [4] or the internal transcribed spacer (ITS) region [5,6].

This study focused on two economically important and closely related downy mildew species, *Pseudoperonospora cubensis* [(Berkeley & M. A. Curtis) Rostovzev] and *P*. *humuli* (Miyabe and Takah., Wilson) [3]. *Pseudoperonospora cubensis* has a relatively wide host range for a downy mildew pathogen [7], afflicting members of the family Cucurbitaceae worldwide, with the most economically important hosts being cucumber (*Cucumis sativus)*, cantaloupe and muskmelon (*Cucumis melo)*, squash and pumpkin (*Cucurbita pepo*, *C*. *maxima and C*. *moschata*) and watermelon (*Citrulus lanatus*) [8]. *Pseudoperonospora humuli* infects hop (*Humulus lupulus*), causing a reduction in hop yield and quality, as well as potential death of the perennial root system [9]. The distinction between these two species has been challenged, as they did not differ consistently in morphology or ITS region sequence [10]. However, further genetic analyses support that the two species are distinct [3,11]. Despite this, only one single nucleotide polymorphism (SNP) has been previously identified that consistently differentiates the two species [3,12] and host range studies have been variable [3,13].

High-throughput sequencing technologies have greatly improved the ability to resolve population genetic structure, develop diagnostic tools and better understand pathogen epidemiology [14]. RNA sequencing (RNA-seq) and genotyping-by-sequencing (GBS) are two such techniques, which can be applied to identify SNPs in transcriptomes as well as genomes. RNA-seq represents expressed genes [15], while GBS samples genomic regions targeted by methylation-sensitive restriction enzymes [16]. RNA-seq has been found to be a very effective technique for SNP discovery [17,18] and can allow for a more accurate functional annotation due to enrichment for expressed genes [19]. However, GBS accesses non-coding DNA, which can contain important regulatory regions controlling phenotypes [16].

The overall purpose of this study was to collect and utilize genomic data to further investigate the genetic differentiation of these two closely related species. In order to accomplish this goal, our first objective was to observe variation spanning the genome and transcriptome between and among isolates of *P*. *cubensis* and *P*. *humuli* using principal components analysis (PCA) [20,21]. Our second objective was to identify SNPs between the species. Our final objective was to annotate the genes containing these SNPs as these genes may be important in host-specificity pathways and could be useful targets for pathogen detection and identification [22,23].

## Results

### Sequencing and alignment

Reduced-representation libraries of *P*. *cubensis* and *P*. *humuli* isolates were sequenced using RNA-seq and GBS (Table 1). For the RNA-seq analyses, 15 isolates of *P*. *cubensis* and 18 isolates of *P*. *humuli* were sequenced, while 20 isolates of *P*. *cubensis* and 18 isolates of *P*. *humuli* were sequenced using GBS (Table 1). The sequencing and alignment results are shown in Table 2.

**Table 1:** Isolates sequenced using RNA-seq and GBS.

Organism	Strain	Host	Year	Location	RNA-seq^a^	GBS^a^
*Pseudoperonospora cubensis*	CDM12- 45	*Cucumis sativus*	2012	Erie, NY	I	S
*Pseudoperonospora cubensis*	CDM12- 58	*Cucumis sativus*	2012	Seneca, NY	I	I
*Pseudoperonospora cubensis*	CDM12- 60	*Cucumis sativus*	2012	Seneca, NY	I	I
*Pseudoperonospora cubensis*	CDM12- 95	*Cucumis sativus*	2012	Cayuga, NY	S	I
*Pseudoperonospora cubensis*	CDM13- 1	*Cucumis sativus*	2013	Erie, NY	I	I
*Pseudoperonospora cubensis*	CDM13- 2	*Cucumis sativus*	2013	Suffolk, NY	I	I
*Pseudoperonospora cubensis*	CDM13- 3	*Cucumis sativus*	2013	Orleans, NY	I	I
*Pseudoperonospora cubensis*	CDM13- 4	*Cucumis sativus*	2013	Ontario, NY	S	I
*Pseudoperonospora cubensis*	CDM13- 6	*Cucumis melo*	2013	Suffolk, NY	I	I
*Pseudoperonospora cubensis*	CDM13- 8	*Cucumis melo*	2013	Ontario, NY	I	I
*Pseudoperonospora cubensis*	CDM13- 9	*Cucumis sativus*	2013	Cayuga, NY	I	I
*Pseudoperonospora cubensis*	CDM13- 10	*Cucurbita pepo*	2013	Suffolk, NY	I	S
*Pseudoperonospora cubensis*	CDM13- 12	*Cucurbita pepo*	2013	Suffolk, NY	S	I
*Pseudoperonospora cubensis*	CDM13- 13	*Cucurbita pepo*	2013	Albany, NY	I	I
*Pseudoperonospora cubensis*	CDM13- 14	*Cucumis sativus*	2013	Erie, NY	E	I
*Pseudoperonospora cubensis*	CDM12-NC	*Cucumis sativus*	2012	NC	NS	I
*Pseudoperonospora cubensis*	CDM-CA	*Cucumis sativus*	2008	CA	NS	I
*Pseudoperonospora cubensis*	CDM13-NC	*Cucumis sativus*	2013	NC	NS	I
*Pseudoperonospora cubensis*	CDM-PM	*Cucurbita maxima*	2013	NC	NS	S
*Pseudoperonospora cubensis*	CDM-SQ	*Cucurbita pepo*	2013	SC	NS	I
*Pseudoperonospora humuli*	HDM 457E	*Humulus lupulus*	2011	Marion, OR	S	S
*Pseudoperonospora humuli*	HDM 481J	*Humulus lupulus*	2011	Ontario, NY	I	I
*Pseudoperonospora humuli*	HDM 482CA	*Humulus lupulus*	2011	Ontario, NY	S	I
*Pseudoperonospora humuli*	HDM490-5	*Humulus lupulus*	2012	Aomori, Japan	I	I
*Pseudoperonospora humuli*	HDM 496SA	*Humulus lupulus*	2012	Hokkaido, Japan	S	S
*Pseudoperonospora humuli*	HDM 498	*Humulus lupulus*	2012	Ehoro, Japan	NS	I
*Pseudoperonospora humuli*	HDM 499AA	*Humulus lupulus*	2013	Marion, OR	I	I
*Pseudoperonospora humuli*	HDM 500BA	*Humulus lupulus*	2013	Marion, OR	I	S
*Pseudoperonospora humuli*	HDM 501AB1	*Humulus lupulus*	2013	Marion, OR	I	S
*Pseudoperonospora humuli*	HDM 502AA	*Humulus lupulus*	2013	Marion, OR	I	I
*Pseudoperonospora humuli*	HDM 503A3	*Humulus lupulus*	2013	Grand Isle, VT	S	I
*Pseudoperonospora humuli*	HDM 503AA	*Humulus lupulus*	2013	Grand Isle, VT	S	NS
*Pseudoperonospora humuli*	HDM 504AB2	*Humulus lupulus*	2013	Grand Isle, VT	S	I
*Pseudoperonospora humuli*	HDM 505–1	*Humulus lupulus*	2013	Ontario, NY	I	I
*Pseudoperonospora humuli*	HDM 506CB	*Humulus lupulus*	2013	Ontario, NY	E	I
*Pseudoperonospora humuli*	HDM 507AA	*Humulus lupulus*	2013	Ontario, NY	I	I
*Pseudoperonospora humuli*	HDM 508AC	*Humulus lupulus*	2013	Ontario, NY	E	I
*Pseudoperonospora humuli*	HDM 509–2	*Humulus lupulus*	2013	Yakima, WA	S	I
*Pseudoperonospora humuli*	HDM 510–1	*Humulus lupulus*	2013	Mazomanie, WI	S	I

^a^ Two sets of analyses were performed, one maximizing the number of SNPs, resulting in the exclusion of isolates with low sequencing depth (filtering priority = max SNPs) and the other retaining isolates at the sacrifice of SNPs (filtering priority = max isolates). I = included in both analyses; S = excluded from the ‘max SNPs’ run but included in the ‘max isolates’ run); E = excluded from both sets of analyses due to missing data; NS = not sequenced.

**Table 2:** Sequencing and alignment results from RNA-seq (n = 33) or GBS (n = 38) analysis.

	RNA-seq	GBS
Total reads	138 million	238 million
Total barcoded reads	100 million	81 million
Barcoded reads aligned to reference^a^	75 million	17 million
Average barcoded aligned reads per isolate	2,272,727	447,368
Total SNPs^b^	140,828	240,841

^a^ The *Pseudoperonospora cubensis* reference genome from Savory et al. (2012) and Burkhardt et al. (2015).

^b^ Total SNPs called using the GATK pipeline (RNA-seq) or Tassel (GBS), prior to filtering using VCFtools.

In order to ensure that the *P*. *cubensis* reference genome [24,25] would be appropriate for alignment of sequences from both species, the percentage of reads aligned to the reference genome for each isolate was calculated and the values were averaged separately for *P*. *cubensis* isolates and *P*. *humuli* isolates. For RNA-seq, 75% and 70% of reads from *P*. *cubensis* and *P*. *humuli* isolates, respectively, aligned. For GBS, 13% and 15% of reads from *P*. *cubensis* and *P*. *humuli* isolates, respectively, aligned to the *P*. *cubensis* reference genome. Thus, reads from *P*. *cubensis* isolates did not align better overall than *P*. *humuli* isolates (S1 Fig).

Table 2 shows that prior to filtering, the average number of barcoded, aligned reads per isolate was on average, 7.6 times higher for the RNA-seq data than for GBS. Overall alignment was 4.2-fold higher for RNA-seq (75 million reads) than for GBS (17 million reads; Table 2). However, GBS had a more even and consistent read depth among isolates, with a standard deviation of aligned reads of 206,898 versus 1.8 million for RNA-seq. Because 8 of the 11 isolates removed from the RNA-seq max-SNPs analysis were *P*. *humuli*, this may be a consequence of high levels of polyphenols from the hop host plant, which may have impacted the library production or fidelity of the sequencing reactions. For both RNA-seq and GBS, differences in sequencing depths between isolates may have been due to differing amounts of plant chemical inhibitors. Finally, GBS produced 71% more total SNPs (240,841) than RNA-seq (140,828; Table 2).

RNA-seq and GBS data contained little host contamination, with the greatest quantity being 1.89% contamination of quality-filtered reads from cucumber for the *P*. *cubensis* RNA-seq data (S1 Table).

### Impacts of filtering strategy: maximizing SNPs retained versus isolates retained

Both RNA-seq and GBS data were filtered in two ways. First, individuals were removed if they contained more than 90% missing data. This resulted in a maximum number of SNPs for downstream analysis (max SNPs). In order to retain more isolates, at the sacrifice of SNPs, (max isolates) the data were also filtered by retaining all isolates with the exception of 3 isolates from the RNA-seq analysis that yielded less than 100,000 total reads (Table 1 and S1 Fig).

For the max SNPs filtering strategy, 1,290 bi-allelic filtered SNPs (0.9% of total SNPs) were retained from the RNA-seq data, while 11,922 (5% of total SNPs) were retained from the GBS data for downstream analysis (S2 Table). Because of the relatively low sequencing read depth of some individuals in the RNA-seq data set, only 19 of 34 isolates (56%) were used for principle components analysis (PCA), while 31 of 38 isolates were retained in GBS analysis (82%; see S2 Table). However, for the isolates retained for PCA, the RNA-seq data had a 63% higher mean read depth per individual, 2.9-fold less missing data per individual and 6.9-fold less missing data per site than the GBS data (S2 Table).

For the max isolates filtering strategy, 30 of 33 sequenced individuals were included in the RNA-seq analyses, which resulted in 135 SNPs (90% reduction). For GBS, all 38 sequenced isolates were maintained in analyses, resulting in 5,044 SNPs (58% reduction; see S2 Table). For the isolates retained for PCA, the RNA-seq data had a 77% higher mean read depth per individual, 2.1-fold more missing data per individual and 2.1-fold more missing data per site than the GBS data (S2 Table).

### Population variation

#### *P*. *cubensis* and *P*. *humuli* isolates

**Fig 1:**
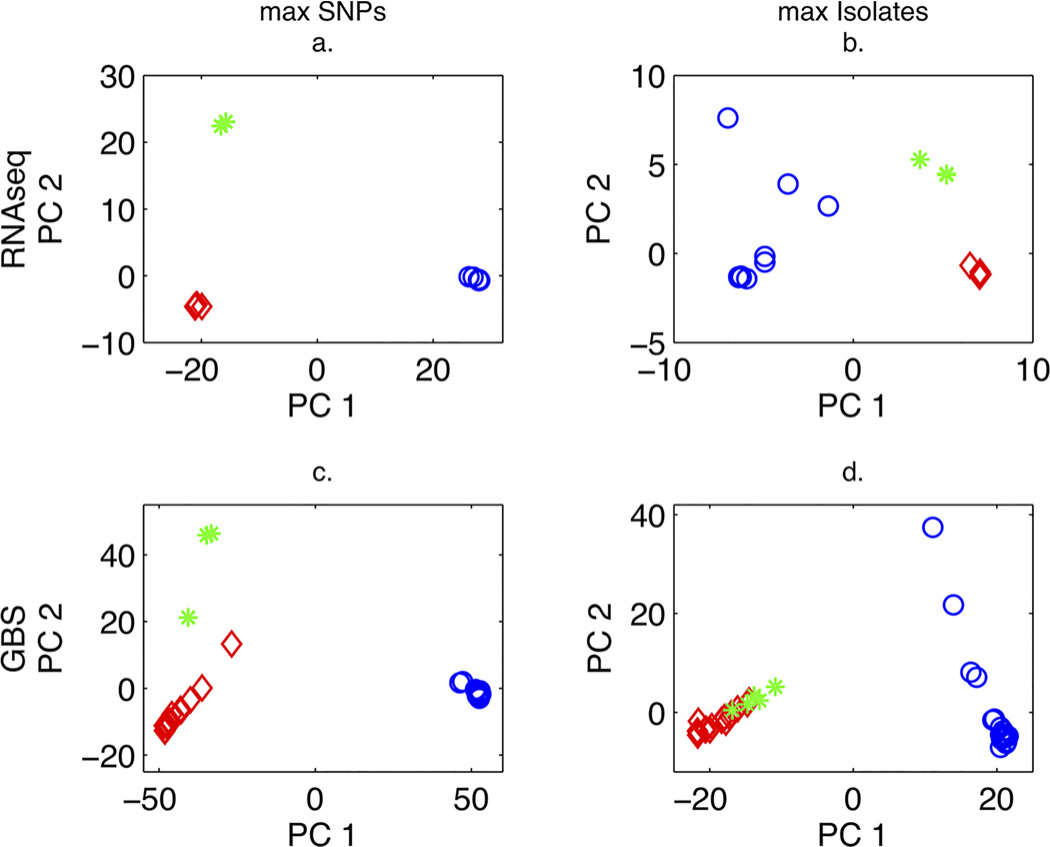
Principal components analysis of RNA-seq and GBS SNP data for *P*. *cubensis* and *P*. *humuli* isolates, maximizing for SNPs or isolates retained a. Includes 19 isolates and 1,290 biallelic SNPs. Blue circles (8) represent *P*. *humuli* isolates. *P*. *cubensis* isolates are represented by red diamonds (9; cucumber and cantaloupe hosts) and green asterisks (2; squash host). b. Includes 30 isolates and 135 biallelic SNPs. Blue circles (16) represent *P*. *humuli* isolates. *P*. *cubensis* isolates are represented by red diamonds (11; cucumber and cantaloupe hosts) and green asterisks (3; squash host). Please note, two of the green asterisks overlap. c. Includes 31 isolates and 11,922 biallelic SNPs. Blue circles (14) represent *P*. *humuli* isolates. *P*. *cubensis* isolates are represented by red diamonds (14; cucumber and cantaloupe hosts) and green asterisks (3; squash host).d. Includes 38 isolates and 5,044 biallelic SNPs. Blue circles (18) represent *P*. *humuli* isolates. *P*. *cubensis* isolates are represented by red diamonds (15; cucumber and cantaloupe hosts) and green asterisks (5; squash and pumpkin hosts).

For both filtering strategies and sequencing techniques, PCA analysis separated isolates of *P*. *cubensis* and *P*. *humuli* (Fig 1). In comparison to the GBS data, the RNA-seq data showed greater separation of isolates of cucumber and cantaloupe hosts from squash hosts among *P*. *cubensis* isolates for both filtering strategies (Fig 1). Increasing the number of isolates included in the analysis, which reduced the number of SNPs in the dataset but increased the number of isolates for RNA-seq by 11 and GBS by 7, did not substantively change the PCA results. PCA plots for GBS contained more isolates than the RNA-seq plots both when the retention of SNPs was prioritized (31 versus 19 individuals) and when retention of isolates was prioritized (38 versus 31; see S2 Table). This difference is partially due to the fact that 5 additional *P*. *cubensis* isolates were sequenced by GBS (Table 1) but also due to the comparatively lower read depth of some isolates using RNA-seq versus GBS (S1 Fig).

Neighbor-joining trees (S2 Fig) for RNA-seq and GBS data from the max SNPs and max isolates filtering strategies corroborated findings from PCA, as the two species grouped separately with 99% or 100% bootstrap support based on max SNP filtered data and 66% to 99% support of max isolate filtered data. Additionally, isolates of *P*. *cubensis* from squash separated from other *P*. *cubensis* isolates with at least 96% support.

#### PCA of *P*. *cubensis* isolates

**Fig 2:**
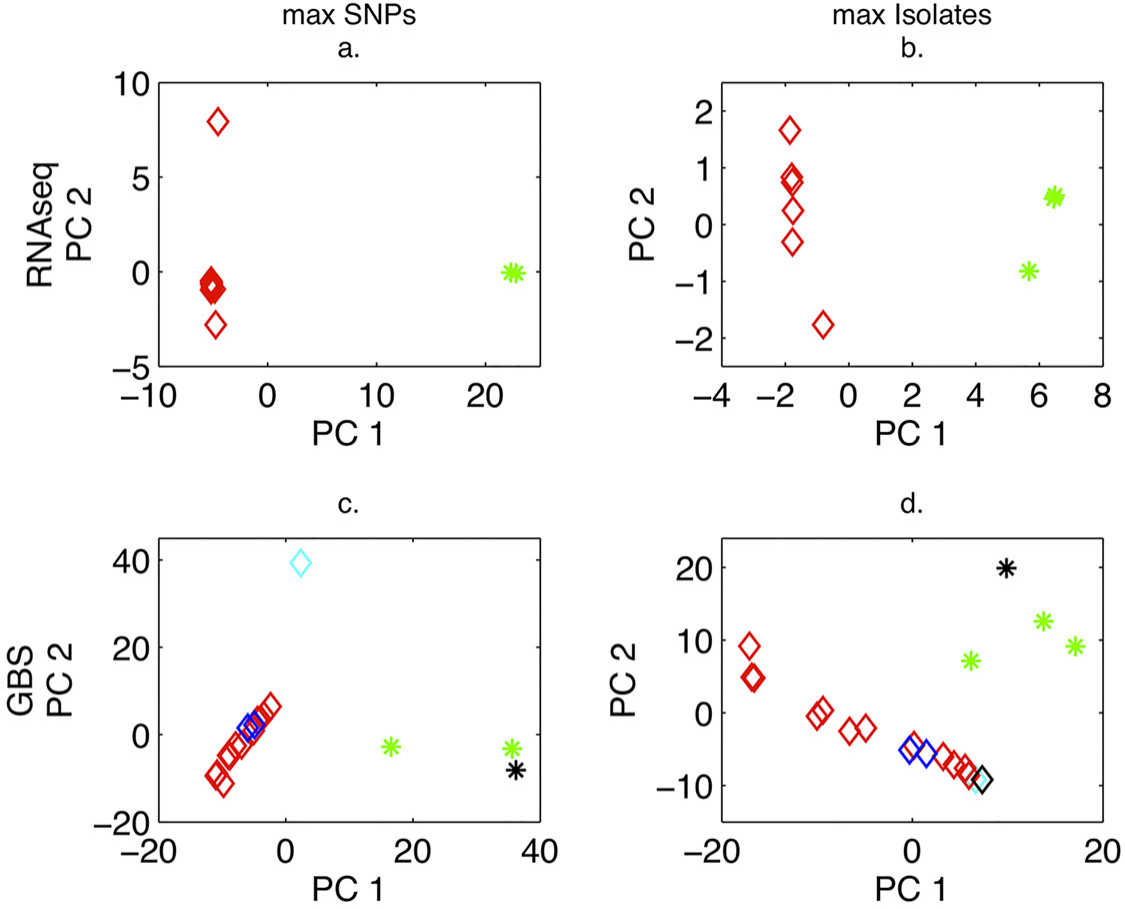
Principal components analysis of RNA-seq and GBS SNP data for *P*. *cubensis* isolates, maximizing for SNPs or isolates retained. a. PCA using 1,290 biallelic SNPs for *P*. *cubensis* isolates only. Red diamonds (9) represent cucumber and cantaloupe isolates and green asterisks (2) represent squash isolates. b. PCA using 135 biallelic SNPs for *P*. *cubensis* isolates only. Red diamonds (11) represent cucumber and cantaloupe isolates and green asterisks (3) represent squash isolates. c. PCA using 11,922 biallelic SNPs for *P*. *cubensis* isolates only. Red diamonds (11) represent cucumber and cantaloupe isolates from NY, and green asterisks (2) represent squash isolates from NY. The black asterisk represents CDM-SQ, a squash isolate from SC. The cyan diamond represents CAcuc2008, a cucumber isolate from CA. The two blue diamonds represent cucumber isolates from NC. d. PCA using 5,044 biallelic SNPs for *P*. *cubensis* isolates only. Red diamonds (12) represent cucumber and cantaloupe isolates from NY, and green asterisks (3) represent squash isolates from NY. The black asterisk represents a squash isolate from SC (CDM-SQ). The black diamond represents a pumpkin isolate from NC (CDM-PM). The cyan diamond represents CDM-CA, a cucumber isolate from CA. The blue diamonds (2) represent cucumber isolates from NC.

PCA of *P*. *cubensis* isolates showed separation of isolates collected on cucumber and cantaloupe hosts from squash hosts for both RNA-seq and GBS (Fig 2). The GBS data contained 5 isolates not included in the RNA-seq data, which were collected from California, North Carolina and South Carolina. When the data were filtered to maximize SNPs, but not when filtered to maximize the number of isolates retained, a separation of the cucumber isolate from California, CDM-CA, from the rest of the cucumber isolates was observed (Fig 2C). For the max SNPs filtering strategy, CDM-CA had 26% missing data versus 5% when the number of isolates was maximized.

#### PCA of *P*. *humuli* isolates

**Fig 3:**
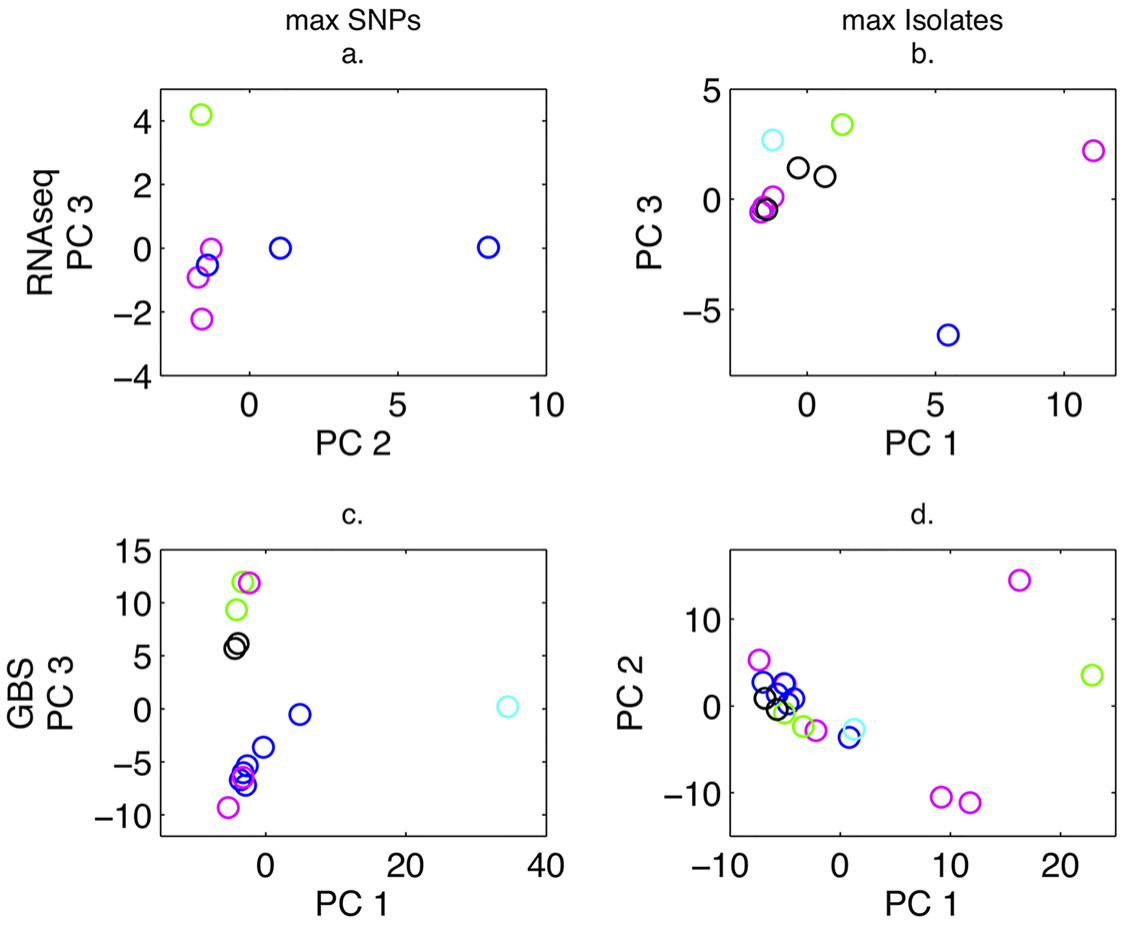
Principal components analysis of RNA-seq and GBS SNP data for *P*. *humuli* isolates, maximizing for SNPs or isolates retained. a. PCA using 1,290 biallelic SNPs for *P*. *humuli* isolates only. The green circle represents the 490–5 isolate from Japan. The blue circles (3) represent isolates from NY. The magenta circles (4) represent isolates from Oregon. b. PCA using 135 biallelic SNPs for *P*. *humuli* isolates only. The blue circles (4) represent isolates from NY. The magenta circles (6) represent isolates from Oregon and Washington. The black circles (3) represent isolates from Vermont. The green circles (2) represent isolates from Japan. The cyan circle represents an isolate from Wisconsin. c. PCA using 11,922 biallelic SNPs for *P*. *humuli* isolates only. The magenta circles (3) represent isolates from OR and WA. The blue circles (6) represent isolates from NY. The black circles (2) represent isolates from VT. The cyan circle represents an isolate from WI. The green circles (2) represent isolates from Japan. d. PCA using 5,044 biallelic SNPs for *P*. *humuli* isolates only. The magenta circles (6) represent isolates from OR and WA. The blue circles (6) represent isolates from NY. The black circles (2) represent isolates from VT. The green circles (3) represent isolates from Japan. The cyan circle represents an isolate from WI.

PCA of *P*. *humuli* isolates from various geographic regions varied depending on the filtering approach and genotyping method (Fig 3). This was most pronounced for RNA-seq data that was filtered for max SNPs versus max isolates. For the GBS data, there also was more separation by geographic region when SNPs were maximized. However, there was overall agreement between RNA-seq and GBS results, excluding the RNA-seq plot maximizing SNPs.

### PCA: Selecting SNPs correlated to separating species

The first principle component, separating the two species, represented 86% and 65% of the variance in the data for RNA-seq and GBS, respectively, in the ‘max SNPs’ data used for the following analyses. Testing for correlation to the first principle component using PCA, 994 and 4,231 PCA-correlated SNPs were selected for RNA-seq and GBS, respectively. These PCA-correlated SNPs were found to contain the expected SNPs containing no missing data (388 and 975 SNPs for RNA-seq and GBS, respectively.)

### Annotation of unigenes containing PCA-correlated SNPs

**Fig 4:**
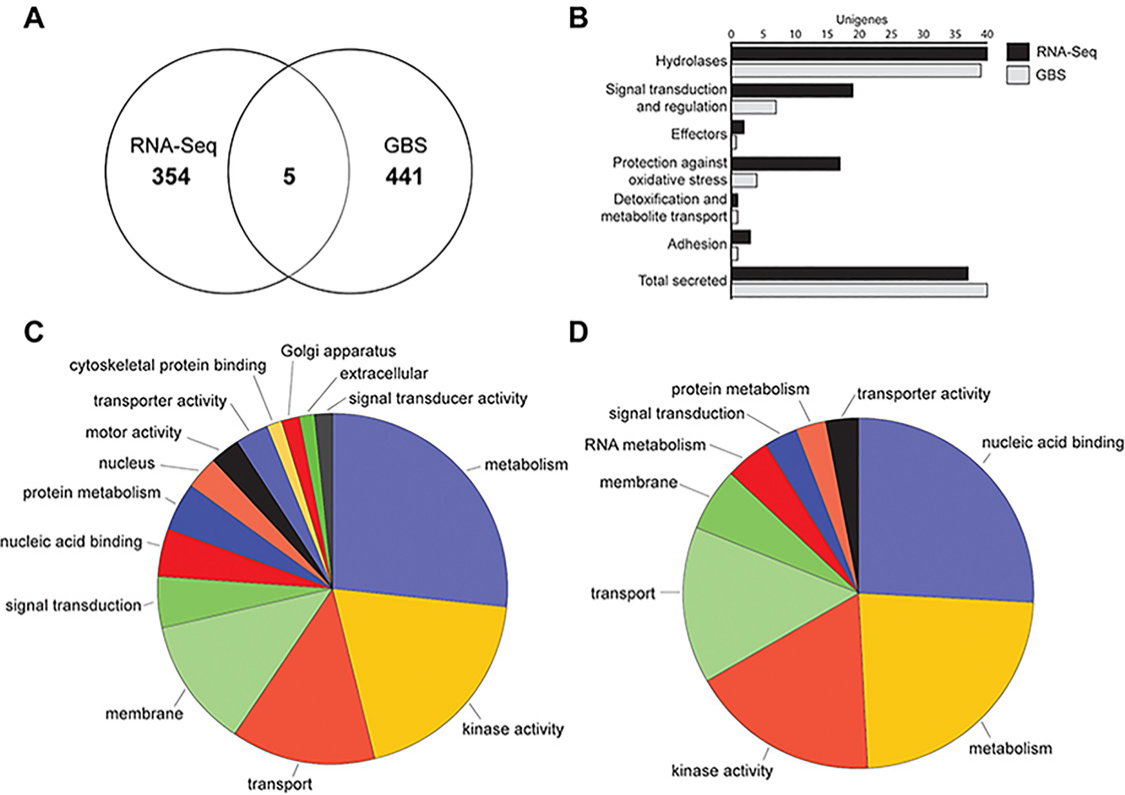
Characterization of unigenes containing PCA-correlated SNPs between *Pseudoperonospora cubensis* and *P*. *humuli* isolates sequenced using RNA-seq and GBS. Using the *P*. *cubensis* reference genome [24,25], unigenes were identified that contained PCA-correlated SNPs from GBS and RNA-Seq data. The number of unigenes identified in each technique, as well as the number of overlapping unigenes are shown in (A). (B) Unigenes classified as putative pathogenicity genes. Gene ontology (GO) of unigenes from (C) RNA-Seq (n = 179) and (D) GBS (n = 89) assigned in terms of the associated biological processes, cellular components and molecular functions.

Eight-hundred total unigenes, or non-redundant and unique genes, were identified which contained PCA-correlated SNPs; 359 were from RNA-seq (containing 994 SNPs), 446 were from GBS (containing 1,547 SNPs) and 5 were overlapping between the two (Fig 4A and S3 Table). A majority of the expressed unigenes lacked sufficient annotation for GO assignments (n = 532) and were excluded from GO analyses. Of the 268 annotated unigenes, 135 were putative pathogenicity genes and 77 were secreted. Of these, 119 were from RNA-seq and 93 were from GBS. These included effectors (n = 3), hydrolases (n = 79), adhesion genes (n = 4), genes involved in signal transduction and regulation (n = 26), protection against oxidative stress (n = 22), and detoxification and metabolite transport (n = 2) (Fig 4B). The three effectors identified included two proteins with RXLR motifs and one elicitin (Table 3). The GO of the 268 total unigenes assigned known functions are shown for RNA-seq (Fig 4C) and GBS (Fig 4D).

**Table 3:** Effector unigenes containing PCA-correlated SNPs.

Unigene ID	Contig	Expressed	Secreted	Source	Effector
PCU_067480	709	Yes	Yes	GBS	RxLR, signal-peptide
PCU_140880	3981	Yes	Yes	RNA-Seq	RxLR, signal-peptide
PCU_163550	6126	Yes	Yes	RNA-Seq	Elicitin, fungal-like

## Discussion

In this study, the results from two reduced-representation library sequencing techniques, RNA-seq and GBS, were used to observe the genetic variation between and among isolates of two closely related obligate biotrophic plant pathogens, *Pseudoperonospora cubensis* and *P*. *humuli*. Although this study examined a limited number of isolates from each species, the PCA results support the findings of previous studies and implement sequencing techniques spanning the genome and transcriptome in order to enhance the resolution of our current understanding of these pathogens’ population variation.

The PCA results using RNA-seq and GBS analysis corroborate earlier conclusions, which were based on a relatively small number of genomic markers, that although highly genetically similar, *P*. *cubensis* and *P*. *humuli* are distinct species [3,26]. In addition, the population structure of *P*. *cubensis* was previously investigated using five nuclear and two mitochondrial loci [27]. Using these seven genes to characterize 465 *P*. *cubensis* isolates collected world-wide from five cucurbit hosts, six genetic clusters were identified, with lower diversity among isolates from cucumber hosts as compared to other hosts [27]. This finding, along with results of other previous studies [28–30] suggest that *P*. *cubensis* isolates exhibit host specificity. In addition, whole-genome sequencing of isolates from different cucurbit hosts identified two distinct lineages among *P*. *cubensis* isolates, with isolates from cucumber, cantaloupe and pumpkin clustering separately from isolates of squash and watermelon [31]. The present study supports these prior investigations, showing differentiation of squash isolates from cucumber, cantaloupe and pumpkin isolates. The GBS data, which included five isolates of *P*. *cubensis* collected in North Carolina, South Carolina and California, also showed that the host-specific trend was consistent across geographic distance, where isolates from cucumber or squash collected in different states clustered closer to isolates of the same host than to other isolates from the same region. However, the genetic relatedness of pumpkin isolates to cucumber and cantaloupe isolates requires further investigation. Previous work has found that pumpkin isolates are distinct from cucumber and cantaloupe isolates with regard to oospore production [32] and are generally found to be the A2 mating type, in contrast to cucumber and cantaloupe isolates [33,34].

Less is known about the population structure of *P*. *humuli*. Chee et al. (2006) examined 40 isolates from each of Oregon and Washington using random amplified polymorphic DNA (RAPD) and DNA amplification fingerprinting (DAF) markers. Their results suggested that the *P*. *humuli* population in Washington was highly clonal while isolates from Oregon were more diverse and they attributed this difference to sexual reproduction in Oregon [35]. The diversity and relatedness of US populations outside of the Pacific Northwest had not been examined prior to this study, as hop production has only recently made a resurgence in the northeastern US [36]. Albeit a small sample size, both sequencing strategies used in our study showed that *P*. *humuli* isolates from New York, Vermont and Japan tended to cluster within their region of collection. However, the clustering of isolates from Oregon and Washington was not consistent and depended on how SNPs were filtered and genotyping method. Future studies including additional isolates from each region may allow for improved resolution of the genetic variation in *P*. *humuli* populations.

The variation differentiating *P*. *cubensis* and *P*. *humuli* was investigated more closely in order to identify genes potentially important for host specificity and disease pathways. We used a modified procedure from Paschou et al. (2007) to select the SNPs correlated to the first principal component, which was the component most important for separating isolates of *P*. *cubensis* and *P*. *humuli*. Similarly, previous studies have used RNA-seq data to identify genes containing polymorphisms potentially important to specific phenotypes; for instance, two alfalfa genotypes with differing cell wall composition [37], two soybean cultivars with differing drought resistance [38] and two *Brassica* species commonly crossed to produce hybrid progeny heterotic for yield [39].

When comparing sequencing data from *P*. *cubensis* and *P*. *humuli*, potential species-specific SNPs were found in 800 total unigenes, with the unigenes from RNA-seq and GBS overlapping for only 5 unigenes. These 800 unigenes represent approximately 2.9% of the 27,591 gene models predicted in the *P*. *cubensis* reference genome used for alignment and annotation [25]. However, similar to previous studies [22], 67% of these total unigenes had no known function. Four of the five SNPs identified by both RNA-seq and GBS were located in unigenes with no known function. However, one of these overlapping unigenes was identified as a secreted signal peptide, and may therefore, be important in pathogenicity pathways [40].

The remaining 268 annotated unigenes containing PCA-correlated SNPs were largely represented by genes involved in metabolism, kinase activity and transport for both RNA-seq and GBS. For GBS, genes associated with nucleic acid binding were also highly represented. Genes involved in metabolism, primarily, but also kinase activity, transport and nucleic acid binding, were the most represented functional classifications in previous studies examining whole genome sequencing data from other oomycete plant pathogens, including *Phytophthora sojae* [22], *Phytophthora infestans* [41] and *Phytophthora parasitica* [42].

Putative pathogenicity genes (n = 135) containing PCA-correlated SNPs were identified. Because effectors are secreted by pathogens in order to manipulate their hosts [40], secreted proteins (n = 77) were also identified from the annotated unigenes. Oomycete pathogen effectors have been characterized by a conserved RXLR (*Arg-X-Leu-Arg*, with X representing any amino acid) motif, which facilitates the delivery of the effector into host cells [43]. A similar conserved motif, QXLR, was identified in 29 secreted peptides in a *P*. *cubensis* genome sequence [44]. The more recent *P*. *cubensis* reference genome used in this study was found to contain 271 putative effectors with an XXLR motif, including 125 putative RXLR effectors [25]. Among the unigenes identified to contain potential species-specific SNPs, 3 contained RXLR motifs. The largest number of putative pathogenicity genes were identified as hydrolases, which degrade components of the plant cell wall [22]. Adhesion genes, which facilitate the pathogen’s entry into host tissue, and genes involved in signal transduction and regulation important for host-pathogen recognition processes [45] were also found. Finally, genes for protection against plant defenses, such as oxidative stress and toxins [45], were identified. Future studies could validate selected PCA-correlated SNPs identified in this study and investigate the impacts of the SNPs between the two species located in putative pathogenicity genes. This may provide greater understanding of the pathogenicity pathways of these two species. Despite their high genetic similarity, *P*. *cubensis* does not infect hop plants and *P*. *humuli* does not infect cucurbits under natural conditions [3], although each pathogen may infect the other host under laboratory conditions [3,33]. Understanding the mechanisms of non-host resistance may inform disease control strategies, such as the breeding of resistant plants [23]. Future studies could also utilize positive selection analyses of the PCA-correlated SNPs located in exons in order to elucidate the genes important in adaptation to hosts.

In conclusion, the PCA results for the RNA-seq and GBS data support the bifurcation of *P*. *cubensis* and *P*. *humuli*. Our data also support results of previous studies that indicate host lineages exist in *P*. *cubensis*. PCA-correlated SNPs responsible for the genetic separation of the two species were located within unigenes and these genes were annotated, with putative pathogenicity genes identified. The PCA-correlated SNPs identified in putative pathogenicity genes may be useful targets for improved diagnosis and detection strategies. Future studies can utilize the streamlined analyses and included scripts for population studies using SNP data.

## Materials and Methods

### Pathogen isolation, maintenance and inoculation

***Pseudoperonospora cubensis***. Fifteen *Pseudoperonospora cubensis* isolates were collected from 15 different fields with symptomatic cucurbit hosts in New York (NY) (8 counties) during the summers of 2012 and 2013 (Table 1). All isolates were collected by obtaining leaves from diseased plants on privately owned farms with permission of the grower and land owner. Ten samples were from cucumber, two from cantaloupe, two from squash and one from pumpkin. Lina Quesada-Ocampo at North Carolina State University contributed 5 DNA samples for GBS from *P*. *cubensis* isolates collected in North Carolina (3 samples: 2 from cucumber and 1 from pumpkin), South Carolina (1 sample from squash) and California (1 sample from cucumber; Table 1).

To obtain single-lesion *P*. *cubensis* isolates, diseased leaves were placed in a moist chamber overnight to induce sporulation. The resulting sporangia were then used to inoculate seven-day old seedlings of the susceptible cucumber cultivar Straight Eight, which were grown in Cornell potting mix (composed of peat, perlite and vermiculite in a 4:1:1 ratio). Sporangia from a single lesion of each isolate were washed from the leaf and sprayed onto seedlings using an air pressurized sprayer (Nalgene, Rochester, NY). This single-lesion process was repeated three times per isolate to reduce the possibility of a mixed genotype. Inoculated plants were placed in dark moist chambers at 16°C overnight then moved to a greenhouse (23.9°C day, 18.3°C night, and 14 hr light). Once lesions appeared on the cucumber seedlings, the plants were placed in a moist chamber (>90% relative humidity) in the dark at 16°C for 24 to 48 hr until prolific sporulation was observed. Live isolates were stored on cucumber leaves at -80°C.

***Pseudoperonospora humuli***. Nineteen *P*. *humuli* isolates were collected from five states as well as Japan during 2011 to 2013 (Table 1). Hop shoots with signs of systemic infection were collected from hop yards and monosporangial isolates of *P*. *humuli* were derived from infected hop shoots as described previously [46] or by depositing a single sporangium onto a leaf disk using a flow cytometer. For the latter method, leaf disks were cut from greenhouse produced hop plants (cv. Pacific Gem) with a #10 cork borer and the adaxial surface placed onto 1% water agar in a 24-well serological plate. A suspension of sporangia from a given sample of *P*. *humuli*, cultured as described by Mitchell et al. (2011), was loaded into a MoFlo flow cytometer **(**Beckman Coulter, Inc., Brea, CA) calibrated to deliver a single sporangium onto each leaf disk. After deposition of sporangia, the leaf disks were misted with sterile deionized water using an airbrush sprayer and incubated in a growth chamber set to 13°C for 7 to 14 days. Leaf disks bearing sporulating lesions were removed and increased on plants of cultivar Pacific Gem and maintained using the methods of Mitchell et al. (2011).

### Collection of sporangia and extraction of total RNA and genomic DNA

Infected leaves of each single sporangium isolate of each pathogen were washed in 40 mL sterile deionized water to remove sporangia. The sporangial suspension was filtered through eight layers of cheesecloth then concentrated by centrifugation at 1,000 x g for 5 min. RNA was extracted using the RNeasy Plant Mini kit (Qiagen, Valencia, CA) and the DNeasy plant mini kit (Qiagen) for DNA extraction. One 5 mm stainless steel bead and 14 silica beads (400 micron) were added to sporangia. For RNA extraction, 450 μl RLT buffer with 2% polyvinylpyrrolidone and 1% beta-mercaptoethanol was added. Buffer AP1 was added for DNA extraction. Samples were then ground at 30 Hz for 2 min in a tissue lyser (Retsch MM400, Cole-Parmer, Vernon Hills, IL). The remaining extraction process followed manufacturer’s protocols.

### RNA-seq library construction and analysis

For RNA-seq library construction, 5 μg of RNA were treated with RQ1 RNase-free DNase (Promega, Madison, WI) and then purified using Dynabeads® Oligo (dT)_25_ (Life Technologies) following the manufacturer’s protocol. Whole transcriptome amplification was performed using the Quantitect Whole Transcriptome Kit (Qiagen, high-yield reaction for 8 hours). The amplified transcriptome was purified with Ampure beads (Beckman-Coulter, Pasadena, CA) then eluted in 100 μl TE buffer. The cDNA was then fragmented using a Covaris machine for fragmentation to 200–250 bp (Duty Cycle: 10%, Intensity: 5, Cycles per burst: 100–180 second) then cleaned with Ampure beads and eluted with water. Frayed DNA ends were treated with End-Repair Master Mix (Epicentre, Charlotte, NC) then purified with Ampure beads. The dA-Tailing Master Mix (New England Biolabs, Ipswitch, MA) was used to add adenine bases to the 3’ ends of fragments, followed by another Ampure purification. Adapters were then ligated to the fragments using T4 DNA ligase (New England Biolabs), followed by Ampure purification. The fragments linked to adapters were subjected to PCR enrichment using Phusion DNA polymerase (New England Biolabs), primers (prAC: 5’-AAT GAT ACG GCG ACC ACC GAG ATC TAC ACT CTT TCC CTA CAC GAC GCT CTT CCG ATC T-3’, prBC: 5’-CAA GCA GAA GAC GGC ATA CGA GAT CGG TCT CGG CAT TCC TGC TGA ACC GCT CTT CCG ATC T-3’), and 5 μl template in a 50 μl reaction. The PCR program consisted of 30 sec at 98°C, [10 sec at 98°C, 30 sec at 65°C, 30 sec at 72°C] 14 cycles total and 5 min 72°C.

The product of PCR enrichment was checked on a 2% agarose gel. Products were purified with Ampure beads and eluted in water. The DNA concentration was measured using Qubit (Life Technologies, Carlsbad, CA) and library concentrations were normalized to 15 ng and multiplexed. Combined libraries were sent to the Genomics Core Facility, Cornell University, for RNA-seq analysis. Samples were sequenced using Illumina Hi-Seq 100 bp single-end sequencing in a single lane. The short reads are linked to Bioproject PRJNA297046 and also available from the Short Read Archive through the National Center for Biotechnology Information (SRP064277).

RNA-seq raw reads were de-multiplexed, trimmed, and quality filtered using the Fastx-toolkit [47]. The publicly available *P*. *cubensis* genome sequence from isolate MSU-1, collected from Homerville, Ohio [24,25] was used as a reference for alignment of both organisms. The 64.4 Mb genome sequence is estimated to represent 73% of the total genome using the predicted size of 88.22 MB determined by Feulgen analysis [48]. Barcoded reads were mapped to the reference genome using Bowtie version 1.0.0 [49] and Tophat version 2.0.13 [50]. Picard Tools version 1.109 was used to add read groups, mark duplicates and reorder alignment files to match the reference genome (http://broadinstitute.github.io/picard). GATK version 3.2–2 was used to prepare splice junctions within the alignment files using the ‘splitNCigarReads’ tool. The ‘HaplotypeCaller’ with default settings was used for calling SNPs to produce the variant call format (VCF) file [51].

### GBS library construction and analysis

DNA samples were submitted to the Cornell University Institute for Genomic Diversity (IGD) for library preparation and sequencing, following a protocol described previously [16]. In brief, adapters were added to the DNA samples, samples were digested with *Ape*K1 restriction enzyme, followed by adapter ligation. The samples were pooled, PCR-enriched, and purified prior to sequencing by Illumina Hi-Seq 100 bp single-end sequencing in a single lane [16]. The short reads are linked to Bioproject PRJNA297163 and also available from the Short Read Archive through the National Center for Biotechnology Information (SRP064284).

Data were filtered by IGD by first aligning to the *P*. *cubensis* reference genome [24]. Raw sequencing reads were processed with the TASSEL-GBS analysis pipeline by IGD using Tassel version 3.0.166 using default parameters, with the exception of two settings. First, a tag was required to be present 3 times in order to be retained in the pipeline. Second, the read depth of each isolate for each tag was recorded in the "Tags-by-taxa" (TBT) file in order to quantitatively call heterozygotes [16,52]. Likelihood scores for each genotype were calculated using formula 3.8 in [53] and the most likely genotype was assigned [54]. The GBS VCF and “Tags-On-Physical-Map” TOPM files were merged with the *P*. *cubensis* genome annotation files corresponding to the reference genome [24,25] available at the Oregon State University Library archives (dx.doi.org/10.7267/N9TD9V7M)

### Measuring host sequence contamination from total quality reads

To measure the amount of host sequence contamination in the RNA-seq and GBS data, the filtered and barcoded reads were separated by species and reads from each species were concatenated. The resulting fastq files were converted to FASTA format using seqtk (https://github.com/lh3/seqtk.git). The sequencing data of each organism were scanned for the number of read hits to host reference genomes using BLAT [55]. For the *P*. *cubensis* data, a cucumber reference genome was used [56]. For the *P*. *humuli* data, a draft hop genome was provided by John Henning, US Department of Agriculture, Agricultural Research Service. The BLAT output was filtered for unique hits to the host genomes and the percent of unique hits from total quality reads was calculated.

### Filtering VCF files from GBS and RNA-seq

RNA-seq and GBS data were filtered in two ways: maximizing SNPs retained and maximizing isolates retained. The former was important in order to capture the most variation within and between isolates. Data were independently filtered to maximize the number of isolates, in order to evaluate as many sequenced isolates as possible. Results from both filtering strategies are presented.

#### Maximizing SNPs retained in downstream analysis

Biallelic SNPs from RNA-seq and GBS data were filtered separately to a minimum genotype quality (minGQ) of 98% using VCFtools version 0.1.12a [57] then converted to PLINK format using PLINK version 1.07 [58] (http://pngu.mgh.harvard.edu/purcell/plink/) in order to determine isolates for exclusion from analysis if they had 90% missing data per individual (0.9 = mind), 20% missing data per marker (0.2 = geno) or less than a minimum minor allele frequency of 1% (maf = 0.01). Using VCFtools, the data were then filtered using the following parameters (after excluding the recommended isolates): 2 maximum and minimum alleles, minGQ of 98%, minimum minor allele frequency of 1% (maf = 0.01) and 10% missing data per marker (max-missing = 0.9). Depth and missing data were calculated for each site and individual using VCFtools. Data filtered using this strategy are henceforth referred to as “max SNPs”.

#### Maximizing isolates retained in downstream analysis

Biallelic SNPs from RNA-seq and GBS data were filtered separately using VCFtools with the same parameters as above. However, isolates recommended for exclusion by PLINK (in the filtering strategy, max SNPs, described above) were retained. For the RNA-seq data, 3 isolates were excluded, as the inclusion of these isolates greatly reduced the number of quality SNPs. For GBS, all sequenced isolates were retained in the downstream analysis. The quality of these files was assessed as described above. Data filtered using this strategy are henceforth referred to as “max isolates”.

If PCA results were conflicting for specific isolates when the RNA-seq and GBS data when the two filtering strategies were compared, the missing data statistics were examined for those isolates specifically and noted.

### Observing population variation

#### Principle components analysis

The VCF files were converted to genotype files then numeric matrices as described in [21] using custom scripts (S1 Scripts). Missing genotype data were filled randomly with numeric genotype calls, which is the most conservative approach [21].

The resulting matrices were passed into a MATLAB PCA routine. Twelve variations of the PCA were completed, with the RNA-seq and GBS data from each of the two filtering strategies (max SNPs and max isolates) repeated for the following: *P*. *cubensis* and *P*. *humuli* isolates together, *P*. *cubensis* isolates separately and *P*. *humuli* isolates separately.

#### Neighbor-joining trees

Single nucleotide polymorphisms from RNA-seq and GBS data for each filtering strategy (max SNPs and max isolates) were converted to a tab delimited text file using VCFtools and concatenated into a FASTA alignment file with a Perl script [59]. Neighbor-joining trees [60] were constructed in Mega6 [61] using the concatenated SNP sequences.

### PCA: Selecting SNPs correlated to separating species

For the following tests, we used the filtered VCF files generated when maximizing the SNPs retained (max SNPs).

#### Selecting SNPs correlated to separating species

Each SNP was scored in a process similar to that previously described [21]. The primary difference between the two processes was that the Paschou et al. (2007) process identified SNPs correlated to an estimated number of principal components accounting for a desired proportion of variance in the data, whereas the current study tested for correlation to the first principal component in the data, which was largely responsible for separating the two species. These SNPs are heretofore referred to as “PCA-correlated SNPs.” In order to check whether the PCA-correlated SNPs contained SNPs we would expect to be present, a list of SNPs that differentiated the two species for all sequenced isolates in our dataset was generated using only SNPs for which there were no missing data. For this, a separate script selecting SNPs conserved among all isolates of the same species and differentiating between species was written.

#### Annotation of unigenes containing PCA-correlated SNPs

PCA-correlated SNPs were linked to corresponding unigenes by overlapping the GFF (general feature format) annotation file (specifically, coding regions and mRNA) from the *P*. *cubensis* reference [24] to the GBS and RNA-seq VCF files using the *intersect* function in BEDTools [62] and R [63]. The term unigene is used to describe a sequence predicted to represent a single, non-redundant gene. Gene ontology (GO) annotations were assigned to unigenes in terms of associated biological processes, cellular components and molecular functions using InterProScan [64]. Unigenes lacking sufficient annotation for GO assignments, such as unintegrated single exon genes (SEG) or hypothetical proteins, were excluded from GO analyses. GO classes were grouped into GO-Slim terms using the web tool CateGOrizer v3.218 [65]. Putative pathogenicity genes [22] containing PCA-correlated SNPs were identified using GO assignments.

## Supporting Information

S1 Fig Total barcoded reads from each sequenced isolate aligned to the *Pseudoperonospora cubensis* reference genome from RNA-seq analysis (a) and GBS analysis (b). Isolates in the blue and green boxes, as well as isolates indicated by green arrows were filtered from the principal components analysis (PCA) maximizing SNP output (max SNPs). Isolates in the blue box (only for RNA-seq data) were excluded from the PCA maximizing isolates retained (max isolates).
(PDF)

S2 Fig Neighbor-joining trees of concatenated SNPs for RNA-seq (A and B) and GBS (C and D) and the two filtering strategies, ‘max SNPs’ (A and C) and ‘max isolates’ (B and D). (PDF)

S1 Scripts Scripts for analysis. Scripts are written in Python and MATLAB and saved in zip file format.
(ZIP)

S1 Table Percent host contamination from total barcoded, unaligned reads for RNA-seq and GBS. (DOCX)

S2 Table Depth and missing data statistics from the filtered VCF files from RNA-seq and GBS data and two filtering strategies, where either the maximum number of SNPs or the maximum number of isolates were retained. (DOCX)

S3 Table Unigenes containing PCA-correlated SNPs identified in both RNA-seq and GBS datasets. (DOCX)
