# Supplementary figures and images for: Identification of Genetic Variation between Obligate Plant Pathogens Pseudoperonospora cubensis and P. humuli Using RNA Sequencing and Genotyping-By-Sequencing
Source: PLoS One. 2015 Nov 23;10(11):e0143665. doi: 10.1371/journal.pone.0143665 (PMC4658093; doi:10.1371/journal.pone.0143665)

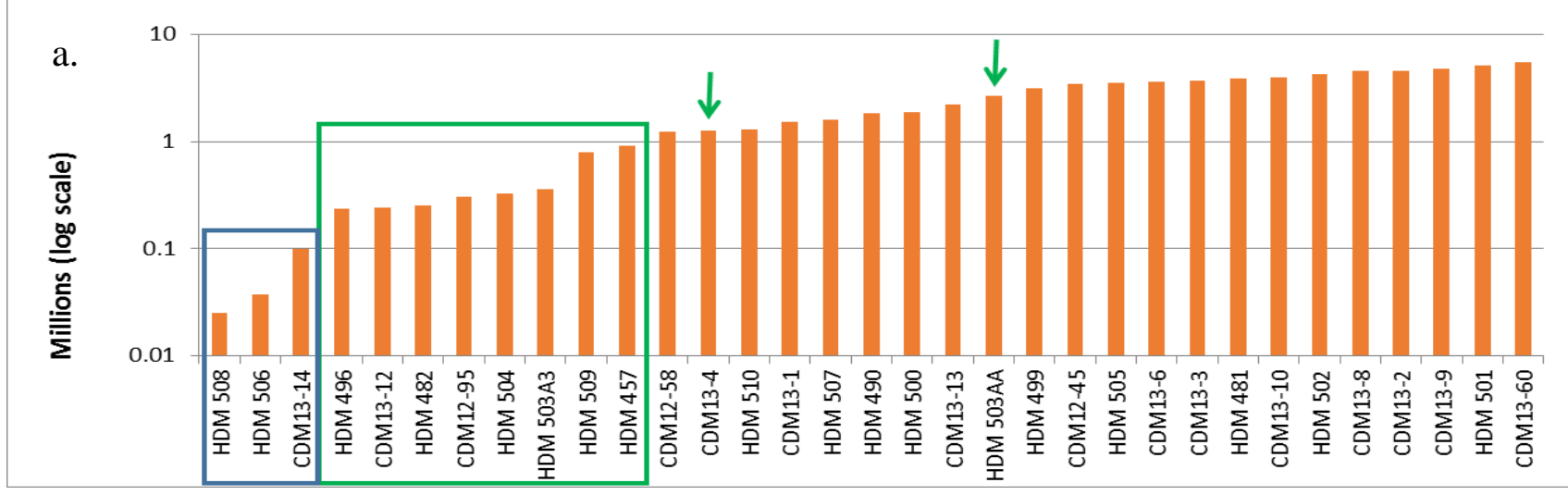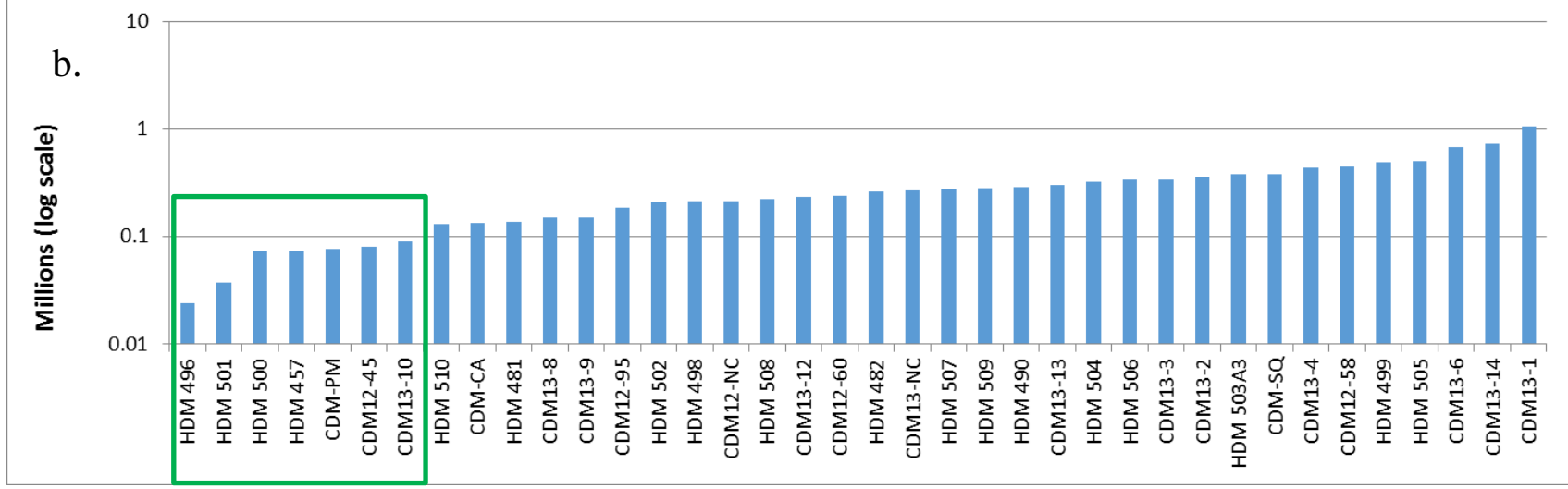

Supplement: S1 Fig — Isolates in the blue and green boxes, as well as isolates indicated by green arrows were filtered from the principal components analysis (PCA) maximizing SNP output (max SNPs). Isolates in the blue box (only for RNA-seq data) were excluded from the PCA maximizing isolates retained (max isolates). (PDF) [file pone.0143665.s001.pdf]
