# Supplementary material for: Identification of Genetic Variation between Obligate Plant Pathogens Pseudoperonospora cubensis and P. humuli Using RNA Sequencing and Genotyping-By-Sequencing
Source: PLoS One. 2015 Nov 23;10(11):e0143665. doi: 10.1371/journal.pone.0143665 (PMC4658093; doi:10.1371/journal.pone.0143665)
Supplement: S2 Fig — (PDF) [file pone.0143665.s002.pdf]

Fig. S2 Neighbor-joining trees of concatenated SNPs

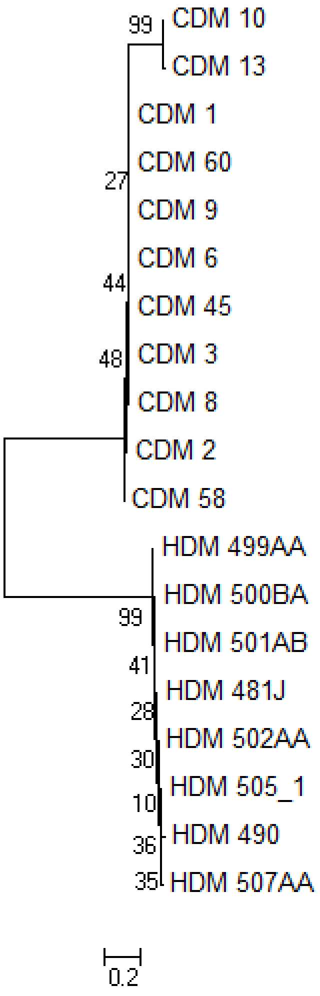

Fig. S2.A Evolutionary relationships of taxa: RNA-seq SNPs filtered for maximum SNP retention.

The evolutionary history was inferred using the Neighbor-Joining method (Saitou & Nei 1987). The optimal tree with the sum of branch length = 1.67639996 is shown. The percentage of replicate trees in which the associated taxa clustered together in the bootstrap test (1000 replicates) are shown next to the branches (Felsenstein 1985). The tree is drawn to scale, with branch lengths in the same units as those of the evolutionary distances used to infer the phylogenetic tree. The evolutionary distances were computed using the Maximum Composite Likelihood method (Tamura et al. 2004) and are in the units of the number of base substitutions per site. The analysis involved 19 nucleotide sequences. Codon positions included were 1st+2nd+3rd+Noncoding. All positions with less than 5% site coverage were eliminated. That is, fewer than 95% alignment gaps, missing data, and ambiguous bases were allowed at any position. There were a total of 1252 positions in the final dataset. Evolutionary analyses were conducted in MEGA6 (Tamura et al. 2014).

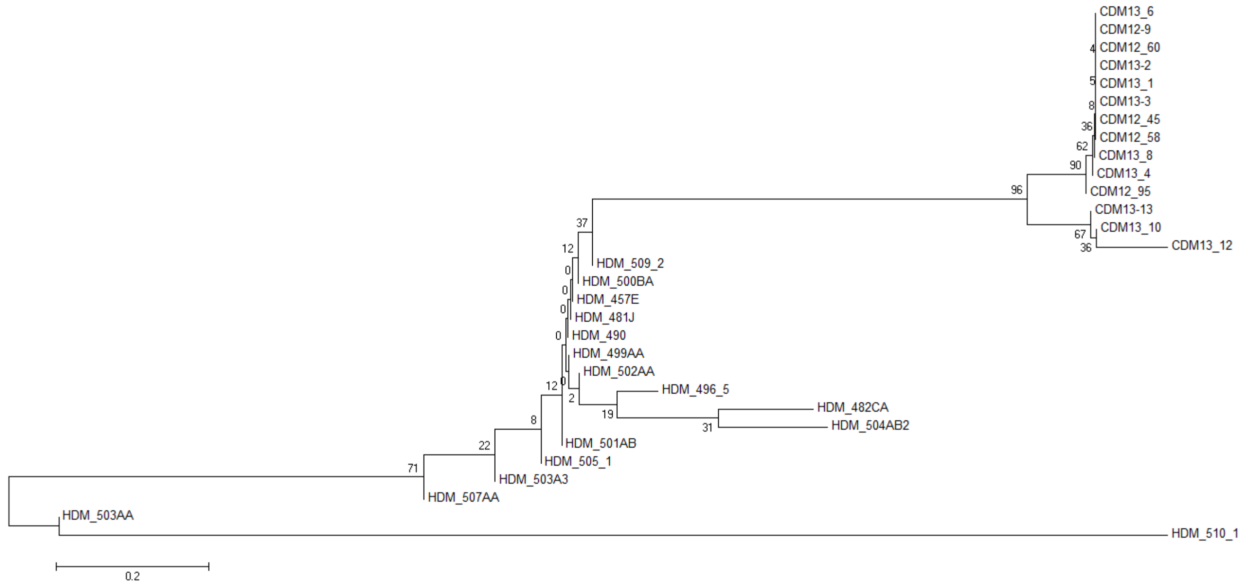

Fig. S2.B Evolutionary relationships of taxa: RNA-seq SNPs filtered for maximum isolate retention.

The evolutionary history was inferred using the Neighbor-Joining method (Saitou & Nei 1987). The optimal tree with the sum of branch length = 3.06710421 is shown. The percentage of replicate trees in which the associated taxa clustered together in the bootstrap test (1000 replicates) are shown next to the branches (Felenstein 1985). The tree is drawn to scale, with branch lengths in the same units as those of the evolutionary distances used to infer the phylogenetic tree. The evolutionary distances were computed using the Maximum Composite Likelihood method (Tamura et al. 2004) and are in the units of the number of base substitutions per site. The analysis involved 30 nucleotide sequences. Codon positions included were 1st+2nd+3rd+Noncoding. All positions with less than 5% site coverage were eliminated. That is, fewer than 95% alignment gaps, missing data, and ambiguous bases were allowed at any position. There were a total of 128 positions in the final dataset. Evolutionary analyses were conducted in MEGA6 (Tamura et al. 2014).

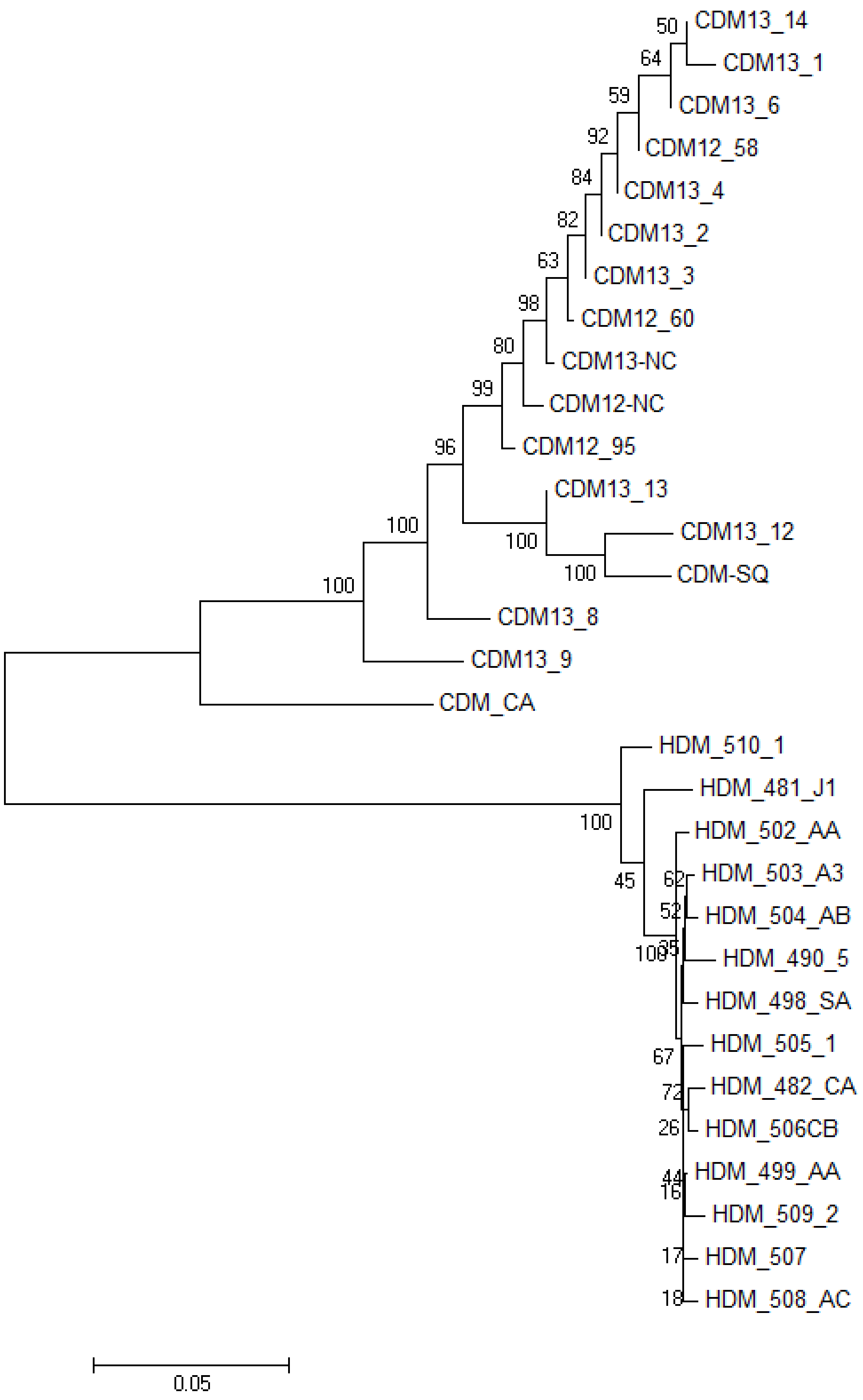

CDM13_14
CDM13_1
CDM13_6
CDM12_58
CDM13_4
CDM13_2
CDM13_3
CDM12_60
CDM13-NC
CDM12-NC
CDM12_95
CDM13_13
CDM13_12
CDM-SQ
CDM13_8
CDM13_9
CDM_CA
HDM_510_1
HDM_481_J1
HDM_502_AA
HDM_503_A3
HDM_504_AB
HDM_490_5
HDM_498_SA
HDM_505_1
HDM_482_CA
HDM_506CB
HDM_499_AA
HDM_509_2
HDM_507
HDM_508_AC
0.05

Fig. S2.C Evolutionary relationships of taxa: GBS SNPs filtered for maximum SNP retention.

The evolutionary history was inferred using the Neighbor-Joining method (Saitou & Nei 1987). The optimal tree with the sum of branch length = 0.58372565 is shown. The percentage of replicate trees in which the associated taxa clustered together in the bootstrap test (1000 replicates) are shown next to the branches (Felenstein 1985). The tree is drawn to scale, with branch lengths in the same units as those of the evolutionary distances used to infer the phylogenetic tree. The evolutionary distances were computed using the Maximum Composite Likelihood method (Tamura et al. 2004) and are in the units of the number of base substitutions per site. The analysis involved 31 nucleotide sequences. Codon positions included were 1st+2nd+3rd+Noncoding. All positions with less than 5% site coverage were eliminated. That is, fewer than 95% alignment gaps, missing data, and ambiguous bases were allowed at any position. There were a total of 11153 positions in the final dataset. Evolutionary analyses were conducted in MEGA6 (Tamura et al. 2014).

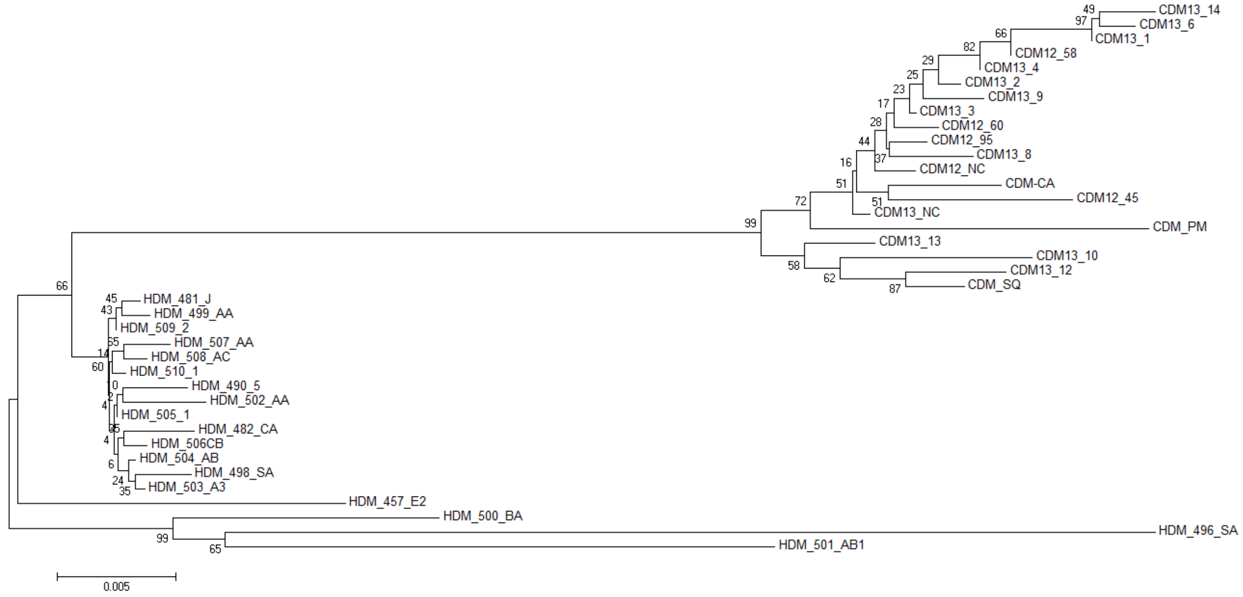

Fig. S2.D Evolutionary relationships of taxa: GBS SNPs filtered for maximum isolate retention.

The evolutionary history was inferred using the Neighbor-Joining method (Saitou & Nei 1987). The optimal tree with the sum of branch length = 0.23338215 is shown. The percentage of replicate trees in which the associated taxa clustered together in the bootstrap test (1000 replicates) are shown next to the branches (Felenstein 1985). The tree is drawn to scale, with branch lengths in the same units as those of the evolutionary distances used to infer the phylogenetic tree. The evolutionary distances were computed using the Maximum Composite Likelihood method (Tamura et al. 2004) and are in the units of the number of base substitutions per site. The analysis involved 38 nucleotide sequences. Codon positions included were 1st+2nd+3rd+Noncoding. All positions with less than 5% site coverage were eliminated. That is, fewer than 95% alignment gaps, missing data, and ambiguous bases were allowed at any position. There were a total of 4593 positions in the final dataset. Evolutionary analyses were conducted in MEGA6 (Tamura et al. 2014).

References

Saitou N, Nei M. 1987. The neighbor-joining method: A new method for reconstructing phylogenetic trees. Mol. Biol. Evol. 4:406-425.

Felsenstein J. 1985. Confidence limits on phylogenies: An approach using the bootstrap. Evol. 39:783-791.

Tamura K, Nei M, Kumar S. 2004. Prospects for inferring very large phylogenies by using the neighbor-joining method. Proc. Nat. Acad. Sci. 101:11030-11035.

Tamura K, Stecher G, Peterson D, Filipski A, Kumar S. 2013. MEGA6: Molecular Evolutionary Genetics Analysis version 6.0. Mol. Biol. Evol. 30: 2725-2729.
