# Supplementary material for: Identification of Genetic Variation between Obligate Plant Pathogens Pseudoperonospora cubensis and P. humuli Using RNA Sequencing and Genotyping-By-Sequencing
Source: PLoS One. 2015 Nov 23;10(11):e0143665. doi: 10.1371/journal.pone.0143665 (PMC4658093; doi:10.1371/journal.pone.0143665)
Supplement: S1 Table — (DOCX) [file pone.0143665.s004.docx]

**Table S1** Percent host contamination from total barcoded, unaligned reads for RNA-seq and GBS.

|  | Species/Host | unique hits | total quality reads | % reads from host |
| --- | --- | --- | --- | --- |
| RNA-seq | *P. cubensis/*cucumber | 1,022,329 | 54,048,200 | 1.89% |
| RNA-seq | *P. humuli/*hop | 211,296 | 45,951,800 | 0.46% |
| GBS | *P. cubensis/*cucumber | 41,619 | 54,426,051 | 0.08% |
| GBS | *P. humuli/*hop | 5,441 | 26,275,783 | 0.02% |
