# Supplementary material for: Identification of Genetic Variation between Obligate Plant Pathogens Pseudoperonospora cubensis and P. humuli Using RNA Sequencing and Genotyping-By-Sequencing
Source: PLoS One. 2015 Nov 23;10(11):e0143665. doi: 10.1371/journal.pone.0143665 (PMC4658093; doi:10.1371/journal.pone.0143665)
Supplement: S2 Table — (DOCX) [file pone.0143665.s005.docx]

**Table S2** Depth and missing data statistics from the filtered VCF files from RNA-seq and GBS data and two filtering strategies, where either the maximum number of SNPs or the maximum number of isolates were retained.

| Maximizing SNPs Retained (max SNPs) | | | | | | |  | Maximizing Isolates Retained (max isolates) | | | |
| --- | --- | --- | --- | --- | --- | --- | --- | --- | --- | --- | --- |
|  | | |  | mean | median | standard deviation |  |  | mean | median | standard deviation |
| **RNA-seq** | | | |  |  |  | **RNA-seq** | |  |  |  |
|  | **(individuals=19, sites= 1,290)** | | |  |  |  |  | **(individuals=30, sites= 135)** |  |  |  |
|  | individual depth^a^ | | | 98 | 103 | 17 |  | individual depth | 120 | 134 | 38 |
|  | site depth^b^ | | | 1865 | 1798 | 534 |  | site depth | 3543 | 3514 | 640 |
|  | site mean depth^c^ | | | 98 | 95 | 28 |  | site mean depth | 121 | 120 | 22 |
|  | missing data (individuals) (%)^d^ | | | 1.4 | 0.6 | 1.9 |  | missing data (individuals) (%) | 7.0 | 0 | 15 |
|  | missing data (sites) (%)^e^ | | | 1.4 | 0 | 2.0 |  | missing data (sites) (%) | 7.0 | 6.6 | 3.0 |
| **GBS** | | | |  |  |  | **GBS** | |  |  |  |
|  | | **(individuals=31, sites= 11,922)** | |  |  |  |  | **(individuals=38, sites= 5,044)** |  |  |  |
|  | | individual depth | | 62 | 59 | 18 |  | individual depth | 92 | 98 | 31 |
|  | | site depth | | 1919 | 1349 | 1460 |  | site depth | 3510 | 3523 | 1502 |
|  | | site mean depth | | 62 | 44 | 47 |  | site mean depth | 92 | 93 | 40 |
|  | | missing data (individuals) (%) | | 4.0 | 2.1 | 5.0 |  | missing data (individuals) (%) | 3.3 | 1.1 | 1.2 |
|  | | missing data (sites) (%) | | 9.7 | 3.2 | 3.6 |  | missing data (sites) (%) | 3.3 | 2.6 | 3.0 |

^a^ Mean depth, or number of reads, per individual (VCFtools output .idepth).

^b^ Depth per site summed across all individuals (VCFtools output .ldepth).

^c^ Mean depth per site averaged across individuals (VCFtools output .ldepth.mean).

^d^ The percentage of missing data for individuals (VCFtools output .imiss).

^e^ The percentage of missing data for sites (VCFtools output .lmiss).
