# Supplementary material for: Identification of Genetic Variation between Obligate Plant Pathogens Pseudoperonospora cubensis and P. humuli Using RNA Sequencing and Genotyping-By-Sequencing
Source: PLoS One. 2015 Nov 23;10(11):e0143665. doi: 10.1371/journal.pone.0143665 (PMC4658093; doi:10.1371/journal.pone.0143665)
Supplement: S3 Table — (DOCX) [file pone.0143665.s006.docx]

**Table S3** Unigenes containing PCA-correlated SNPs identified in both RNA-seq and GBS datasets.

| Unigene ID | Contig | Splice Type | Secreted | RNA-Seq^a^ | GBS^a^ | Putative Function |
| --- | --- | --- | --- | --- | --- | --- |
| PCU_004770 | 18 | No introns | Yes | 6 | 1 | SEG^b^, signal-peptide |
| PCU_076920 | 918 | No introns | No | 6 | 1 | DUF106^c^, transmembrane |
| PCU_111220 | 2163 | Fully-spliced | No | 4 | 1 | hypothetical protein |
| PCU_174420 | 7414 | No data | No | 2 | 4 | hypothetical protein |
| PCU_182850 | 8557 | No introns | No | 1 | 5 | SEG, hypothetical protein |
| ^a^ Total number of PCA-correlated SNPs within the overlapping unigenes.  ^b^ Unintegrated single exon gene.  ^c^ Integral membrane protein of unknown function. | | | | | | |
